# A LOGIC MODEL TO DESCRIBE ADULT PROTECTIVE SERVICES IN THE U.S

**DOI:** 10.1093/geroni/igz038.1859

**Published:** 2019-11-08

**Authors:** Zach Gassoumis, Karl Urban, Gila Shusterman, Stephanie Whittier Eliason

**Affiliations:** 1 Leonard Davis School of Gerontology, University of Southern California, Los Angeles, California, United States; 2 WRMA, Inc, Rockville, Maryland, United States; 3 Administration for Community Living, Washington, D.C., United States

## Abstract

The adult protective services (APS) system in the U.S. serves as an investigative and service delivery system, targeting cases of reported abuse, neglect, and exploitation of older adults and adults with disabilities. APS developed from a piecemeal investigatory system, driven by state and local processes. This system has persisted, with little federal oversight or uniformity. In turn, little systematic information exists about the structure and process of the APS system nationwide. This poster presents an effort to construct a logic model for APS in the U.S., compiled jointly by researchers and practitioners under the auspices of the National APS Technical Assistance Resource Center. The process included initial drafting by six experts from various backgrounds and was reviewed by diverse stakeholders in three iterative rounds of review. Because of the unique nature of APS, the product has a partially non-traditional structure for a logic model, plotting context, inputs/resources, activities, activity metrics, and results. Inputs/resources are plotted across various sources: APS staff, consultative experts, community partners, operational supports, funding for services, and legal and ethical processes. Activities, activity metrics, and results are mapped across three stages of service provision—intake, investigation, and post-investigation services—and at the quality assurance level. The resultant logic model can be used at the national level to drive system change and evaluation; alternatively, it can be customized to specific state/local contexts to enable quality improvement and evaluation efforts. The pursuit of these efforts, coupled with existing national strategies, can contribute to continued system change and evolution within APS.

